# Supplementary material for: Complex‐centric proteome profiling by SEC‐SWATH‐MS
Source: Mol Syst Biol. 2019 Jan 14;15(1):e8438. doi: 10.15252/msb.20188438 (PMC6346213; doi:10.15252/msb.20188438)
Supplement: Supplementary file 8 — Dataset EV7 [file MSB-15-e8438-s008.zip › feature_plots_string/Q9Y2Z9.pdf]

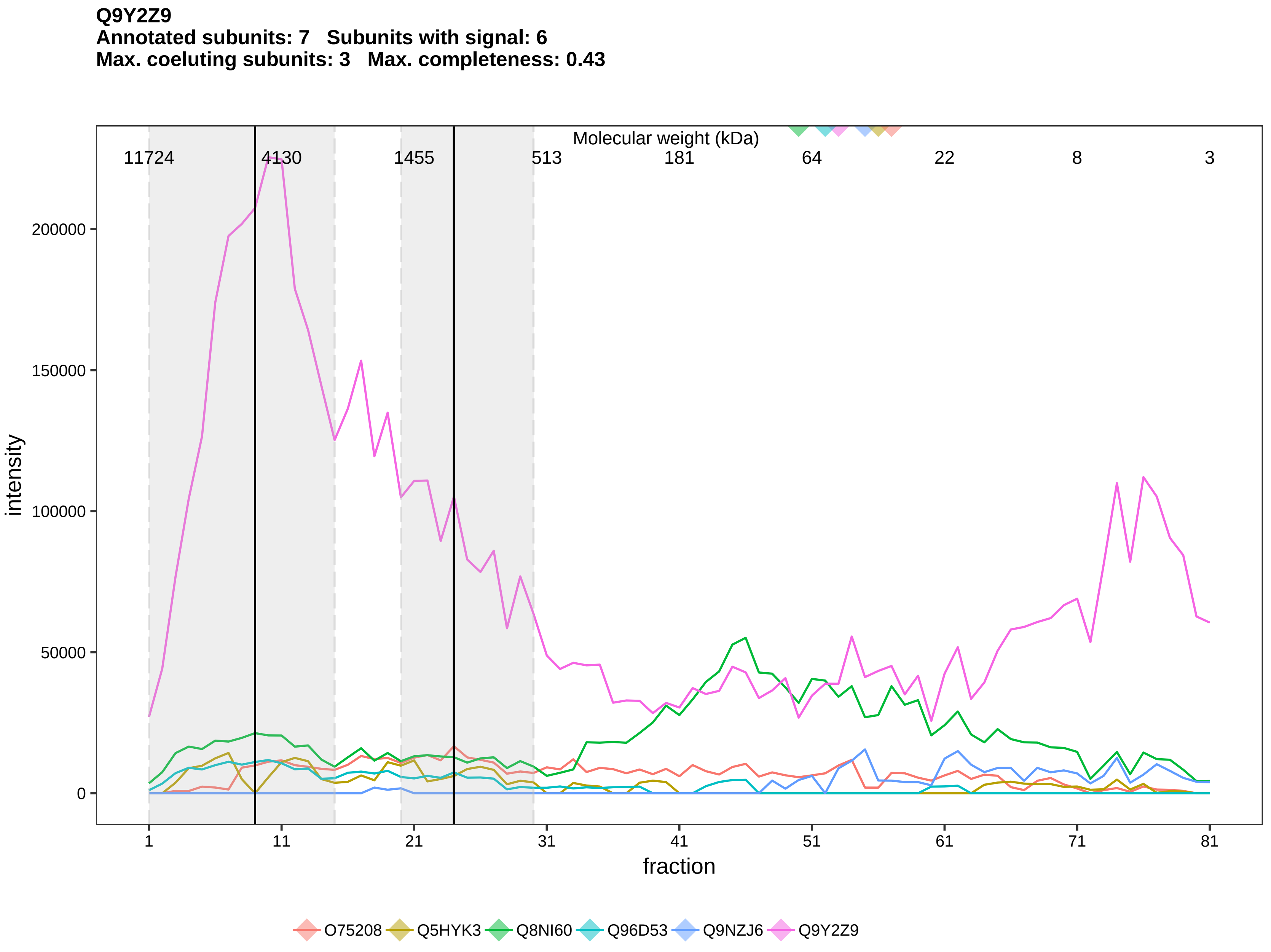

Q9Y2Z9
Annotated subunits: 7 Subunits with signal: 6
Max. coeluting subunits: 3 Max. completeness: 0.43
Molecular weight (kDa)
11724
4130
1455
513
181
64
22
8
3
intensity
200000
150000
100000
50000
0
1
11
21
31
41
51
61
71
81
fraction
O75208
Q5HYK3
Q8NI60
Q96D53
Q9NZJ6
Q9Y2Z9
